# Characterization and assembly of the *Pseudomonas aeruginosa* aspartate transcarbamoylase-pseudo dihydroorotase complex

**DOI:** 10.1371/journal.pone.0229494

**Published:** 2020-03-03

**Authors:** Chandni Patel, Asmita Vaishnav, Brian F. P. Edwards, David R. Evans

**Affiliations:** Department of Biochemistry, Microbiology, and Immunology, Wayne State University School of Medicine, Detroit, Michigan, United States of America; Griffith University, AUSTRALIA

## Abstract

*Pseudomonas aeruginosa* is a virulent pathogen that has become more threatening with the emergence of multidrug resistance. The aspartate transcarbamoylase (ATCase) of this organism is a dodecamer comprised of six 37 kDa catalytic chains and six 45 kDa chains homologous to dihydroorotase (pDHO). The pDHO chain is inactive but is necessary for ATCase activity. A stoichiometric mixture of the subunits associates into a dodecamer with full ATCase activity. Unlike other known ATCases, the *P*. *aeruginosa* catalytic chain does not spontaneously assemble into a trimer. Chemical-crosslinking and size-exclusion chromatography showed that *P*. *aeruginosa* ATCase is monomeric which accounts for its lack of catalytic activity since the active site is a composite comprised of residues from adjacent monomers in the trimer. Circular dichroism spectroscopy indicated that the ATCase chain adopts a structure that contains secondary structure elements although neither the ATCase nor the pDHO subunits are very stable as determined by a thermal shift assay. Formation of the complex increases the melting temperature by about 30°C. The ATCase is strongly inhibited by *all* nucleotide di- and triphosphates and exhibits extreme cooperativity. Previous studies suggested that the regulatory site is located in an 11-residue extension of the amino end of the catalytic chain. However, deletion of the extensions did not affect catalytic activity, nucleotide inhibition or the assembly of the dodecamer. Nucleotides destabilized the dodecamer which probably accounts for the inhibition and apparent cooperativity of the substrate saturation curves. Contrary to previous interpretations, these results suggest that *P*. *aeruginosa* ATCase is not allosterically regulated by nucleotides.

## Introduction

Aspartate transcarbamoylase (ATCase; EC 2.1.3.2) catalyzes the reaction of carbamoyl phosphate (CP) and aspartate to form N-carbamoyl-l-aspartate (CA) and inorganic phosphate [1] a key step in *de novo* pyrimidine biosynthesis. The catalytic subunit or domain of all known ATCases in eukaryotes, prokaryotes and archaea have a molecular mass of approximately 34 kDa and form stable homo-trimers under physiological conditions. Although ATCase is ubiquitous and catalyzes the same reaction, the enzyme from different organisms is remarkably polymorphic, differing in oligomeric structure, composition and regulatory properties.

In mammals [2,3], the first three enzymes of pyrimidine biosynthesis are consolidated on a single 243 kDa polypeptide, called CAD. The multifunctional protein has discrete domains catalyzing the first three steps in *de novo* pyrimidine biosynthesis, carbamoyl phosphate synthetase, aspartate transcarbamoylase and dihydroorotase. Each domain, either isolated from controlled proteolytic digests or subcloned and expressed in *E*. *coli*, was found to be fully functional. *Drosophila melanogaster* [4] and the protist, *Dictyostelium discoideum* [5], have the same structural organization.

The ATCase from fungi such as *Saccharomyces cerevisiae* [6] *Neurospora crassa* [7,8] *and Chaetomium thermophilum* [9] are also multifunctional with a similar structural organization to CAD. The proteins have glutamine dependent-carbamoyl phosphate synthetase and aspartate transcarbamoylase activity, but lack DHOase activity. However, the protein has a pseudo-DHO domain (pDHO) that has the same size and is homologous to the active DHOase of other organisms but lacks the zinc ligands and substrate binding residues. The structures of the *C*. *thermophilium* ATCase and pDHOase domains have recently been solved [9]. The X-ray structure of pDHO was found to have a very similar fold as the active DHOase from other organisms.

Bethell and Jones [10] have identified three major classes of bacterial ATCases based on their size and regulatory properties. The enzyme from *E*. *coli*, the archetypal ATCase, was classified as Class B. It is an allosteric 300 kDa dodecamer that binds aspartate cooperatively and is regulated by nucleotides. The enzyme can be dissociated into two catalytic trimers (C3) and three regulatory dimers (R2) [11–14]. The catalytic subunits catalyze the formation of carbamoyl aspartate but are unregulated. The regulatory subunits bind the allosteric inhibitors, CTP and UTP and the activator ATP. X-ray studies [15–17] showed that two catalytic trimers are stacked above each other in nearly eclipsed configuration and are held together by three regulatory dimers, which are clustered around the periphery of the molecule.

Class C ATCases are the smallest with a molecular mass of approximately 100 kDa. These enzymes, such as *Bacillus subtilis* ATCase [18], are unregulated trimers that do not associate with regulatory subunits or any other enzyme in the pyrimidine pathway. X-studies [19] of *B*. *subtilis* ATCase showed that its tertiary structure is very similar to the catalytic subunit of *E*. *coli* ATCase.

The Class A enzymes are the largest ATCases with a molecular mass close to 500 kDa. Class A1 ATCases are comprised of active ATCase and DHOase subunits, e.g. *Streptomyces griseus* [20] and *Aquifex aeolicus*[21–23]. X-ray studies [23] of *A*. *aeolicus* ATCase-DHOase dodecameric complex have shown that two ATCase trimers are held together by three DHOase dimers, an oligomeric structure reminiscent of the 32 symmetry of *E*. *coli* ATCase, where the regulatory dimers are replaced by dimers of DHOase subunits. The class A2 enzymes, such as *Pseudomonas* ATCases, have a similar structure organization but have active ATCase and an inactive pDHO subunits homologous to the domain of the yeast multifunctional protein, ura2.

The first Class A2 ATCase was purified by Adair and Jones [24] from *Pseudomonas fluorescens*, who found that the protein was a dimer composed 180 kDa subunits and that it was inhibited by ATP, CTP and UTP. In view of the unusual subunit structure that suggested that the catalytic unit is not trimeric, we re-examined the size, composition and regulation of *P*. *fluorescens* ATCase [25]. In this study, isolated *P*. *fluorescens* ATCase was found to be a 460 kDa dodecamer composed of six copies of a 34 kDa catalytic trimer and six copies of a 45 kDa subunit of unknown function. The function of the isolated subunits could not be ascertained because they could not be separated without denaturation and loss of ATCase activity. However, the 34 kDa polypeptide was identified as ATCase catalytic chain by affinity labelling of the active site with the competitive inhibitor, [^14^C]-pyridoxal phosphate followed by sodium borohydride reduction. Schurr et al.[26] subsequently sequenced the 45 kDa subunit and showed that it was a pDHO.

We then investigated the ATCase from *P*. *aeruginosa* [27] and found that it had the same subunit structure and putative regulatory properties. All oxy- and deoxynucleotide triphosphates strongly inhibited the enzyme. Free GTP and ATP, but not the Mg^2+^ATP, inhibited the enzyme. It is difficult to reconcile these results with the proposed function of allosteric regulation of ATCases, namely, to balance the levels of pyrimidines and purines. Alternately, the instability of the catalytic trimer may also account for nucleotide inhibition. Moreover, the extraordinarily cooperative aspartate saturation curves (Hill coefficient > 10) appear unrealistic with common understanding of homotropic interactions where the upper limit, rarely achieved, is equal to the number of subunits.

We report here the overexpression and purification of *P*. *aeruginosa* ATCase and pDHO, and determination of oligomeric states of individual proteins to better understand the assembly of the dodecamer. Unlike the ATCase from other organisms, the catalytic unit is monomeric and only becomes a catalytically active trimer when it associates with pDHO. The instability of the catalytic subunit may also explain the unusual inhibition by nucleotides.

## Materials and methods

### Materials

Reagents including aspartate, carbamoyl phosphate, antipyrine, and diacetyl monooxime were obtained from Sigma, pETite vector and *E*. *coli* Hi-Control BL21(DE3) cells from Lucigen, and BS3 (bis (sulfosuccinimidyl) suberate) cross-linker from Thermo Fisher Scientific.

### Expression and isolation of the recombinant proteins

The genes encoding *P*. *aeruginosa* ATCase and pDHO were cloned separately into pETite (Lucigen), an expression vector that incorporates a 6xHis tag on the amino end of the recombinant protein. The resulting constructs were transformed separately into *E*. *coli* Hi-Control BL21(DE3) cells. The transformants were grown at 37°C in LB medium until OD_600_ of 0.6 and induced with 0.1 mM IPTG overnight (16–20 hours) at 20°C. The cells were harvested and resuspended in 50 mM sodium phosphate and 0.5 M NaCl, pH 7.5, and passed twice through a French press. The cell lysate was centrifuged at 12,000 x g for 30 min at 4°C. The supernatant was applied to 5.0-mL Ni^2+^ Probond affinity column (Invitrogen). The column was washed with 10 column volumes and the proteins were eluted in 5-mL aliquots with 250 mM imidazole in the same buffer. The fractions were analyzed by electrophoresis on 12% SDS-PAGE gels[28]. Pure protein fractions were pooled and concentrated in the Amicon Ultra-15 centrifugal filter units (EMD Millipore). Protein concentrations were then determined by the Lowry method [29] using bovine serum albumin as a standard.

### Enzyme assay

ATCase activity was measured by the colorimetric method as previously described by Prescott and Jones[30, 31]. The reaction mixture to measure carbamoyl aspartate saturation of ATCase consisted of 8 mM aspartate, 50 mM Tris/acetate buffer, pH 8.3, and 1–4 μg of purified ATCase and a stoichiometric amount of pDHO and variable (0–8 mM) carbamoyl phosphate in a total volume of 1 mL. The reaction mixture for aspartate saturation consisted of 10 mM carbamoyl phosphate, 50 mM Tris/acetate buffer, pH 8.3, 1–4 μg of purified ATCase and a stoichiometric amount of pDHO and variable (0–15 mM) aspartate in a total volume of 1 mL. Samples were pre-incubated at 37°C for 1 min. The reaction was then initiated by the addition of carbamoyl phosphate or aspartate, 0–15 mM, and allowed to proceed for 2 min. The reaction was quenched by the addition of 1 mL of 5% acetic acid. Then 2 mL of color mix containing antipyrine/diacetyl monooxime in a 2:1 ratio was added, and the assay mixture was heated at 60°C for 60 min for color development. The carbamoyl aspartate generated by ATCase is converted into a yellow chromophore and the absorbance was measured at 466 nm.

### Size exclusion chromatography

The molecular mass of the recombinant proteins was determined by size exclusion on an AKTA chromatography system. The Ni^2+^ column purified pDHO, ATCase, and the ATCase-pDHO complex, reconstituted by mixing equimolar amounts of the individual proteins, were applied separately to a Superdex S-200 column equilibrated with 50 mM Tris-HCl, pH 7.5 and 200 mM NaCl. A 2-mL sample of purified protein, 5–10 mg/ml, was applied and eluted with the same buffer at a flow rate of 0.5 mL/min. The protein fractions were analyzed by electrophoresis on 12% polyacrylamide gel. The column was calibrated with a mixture of standard proteins and the mass of the eluted species was determined from plots of log (Mr) versus elution volume (S3 Fig).

### Chemical crosslinking

Purified recombinant ATCase (45 μM) or pDHO (37 μM) in 50 mM sodium phosphate, pH 7.5, 500 mM NaCl, and 5% glycerol was cross-linked for the indicated times with 5 mM bis(sulfosuccinimidyl) suberate. For the ATCase-pDHO complex, ATCase (36 μM) and pDHO (37 μM) in 50 mM sodium phosphate, pH 7.5, 500 mM NaCl, and 5% glycerol were crosslinked as described above. The reaction was quenched by adding 10 μL of 1 M Tris-HCl, pH 8. Cross-linked species were analyzed by SDS electrophoresis on 7.5% or 4–20% gradient polyacrylamide gel.

### CD spectrometry

The CD spectra of pDHO (1.8 μM), ATCase (2.67 μM), and the ATCase-pDHO complex (1.8 μM) prepared by mixing stoichiometric amounts of pDHO and ATCase, were obtained at room temperature in the wavelength range of 185–300 nm using a Jasco CD spectrophotometer with a 1mm quartz cuvette. The proteins were prepared in 5 mM sodium phosphate, pH 8.

### Comparative modeling

The tertiary structure of the *P*. *aeruginosa* pDHO subunit was modeled on the SIB Bioinformatics Research Portal (ExPASy) using the 2.3 Å structure [23] of the dihydroorotase subunit of the *A*. *aeolicus* DHOase-ATCase complex (4BJH.pdb) as a template.

### Controlled proteolysis by elastase

Purified recombinant ATCase was subjected to controlled proteolysis at 37°C at a protein-to-elastase ratio (wt/wt) of 250. The reaction mixture consisted of 160 μg of ATCase and 0.64 μg of elastase per 160 μL in 50 mM Tris-HCl, pH 8, and 200 mM NaCl with or without 100 μM PALA. Samples were taken periodically over two hours and quenched by adding 5 μL of sample buffer consisting of 50 mM Tris-HCl, pH 6.8, 2% SDS, 10% glycerol, 1% beta-mercaptoethanol, and 0.02% bromophenol blue, and by heating for 5 min at 100°C. The samples were analyzed by electrophoresis on SDS 12% polyacrylamide gel.

### Thermal shift assay

The assay was performed using the 96-well PCR microplate (Applied Biosystems) and SYPRO Orange dye (Invitrogen). Each well contained 1 μL of purified recombinant protein, ATCase (2.5 mg/mL), pDHO (2.5mg/mL), or ATCase-pDHO complex (1.25 mg/mL), 1 μL of 500X SYPRO Orange dye (5000X diluted into water), and 23 μL of buffer containing 50 mM Tris pH 7.5 and 200 mM NaCl. The microplate was covered with a transparent adhesive seal and centrifuged for 30 seconds at 1000 x g before analysis in an Applied Biosystems 7500 Real-Time PCR instrument. The melting temperature was determined using the Applied Biosystems 7500 PCR software.

## Results

### Cloning, expression, and purification of *P*. *aeruginosa* ATCase and pDHO

**Fig 1:**
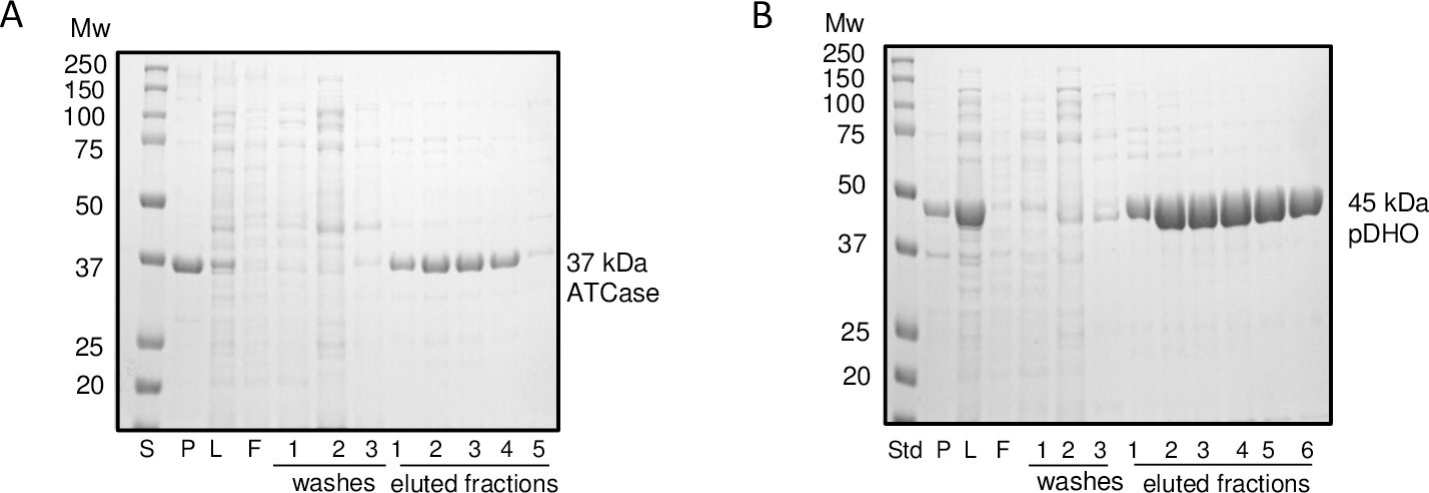
Expression and purification of *P*. *aeruginosa* ATCase and pDHO. The genes encoding *P*. *aeruginosa* ATCase (pyrB) and pDHO (pyrX) were separately cloned into pETite (Lucigen), an expression vector that incorporates a 6xHis tag on the amino end of the recombinant proteins. The resulting constructs were transformed separately into *E*. *coli* Hi-Control BL21(DE3) cells (Lucigen). The transformants were grown at 37°C in LB medium until OD600 of 0.6 and induced with 0.1 mM IPTG overnight (16–20 hours) at 20°C. The cells were harvested and resuspended in 50 mM sodium phosphate and 0.5 M NaCl, pH 7.5, and passed twice through a French press. The cell lysate was centrifuged at 12,000 x g for 45 min at 4°C. The supernatant was applied to 5.0 ml Ni^2+^ affinity column. The column was washed with 10 column volumes with increasing concentration of imidazole up to 100 mM. The proteins were eluted in 5 mL aliquots with 250 mM imidazole in the same buffer. A) ATCase and B) pDHO fractions were analyzed by electrophoresis on 12% SDS-PAGE Laemmli gels [28].

The *pyrB* gene encoding *P*. *aeruginosa* ATCase and *pyrX* encoding pDHO from strain PAO1 were separately amplified by PCR and inserted into the pETite vector, which incorporates a 6x His tag on the amino terminus of the proteins. The resulting constructs were then transformed into *E*. *coli* Hi-Control BL21(DE3) cells. The proteins were expressed and purified as described under “Materials and methods”. Although significant amounts of both proteins formed inclusion bodies, the soluble proteins were both expressed at 40 mg/liter (Fig 1A and 1B, S2 Fig).

### Oligomeric structure of *P*. *aeruginosa* ATCase and pseudo-DHO

**Fig 2:**
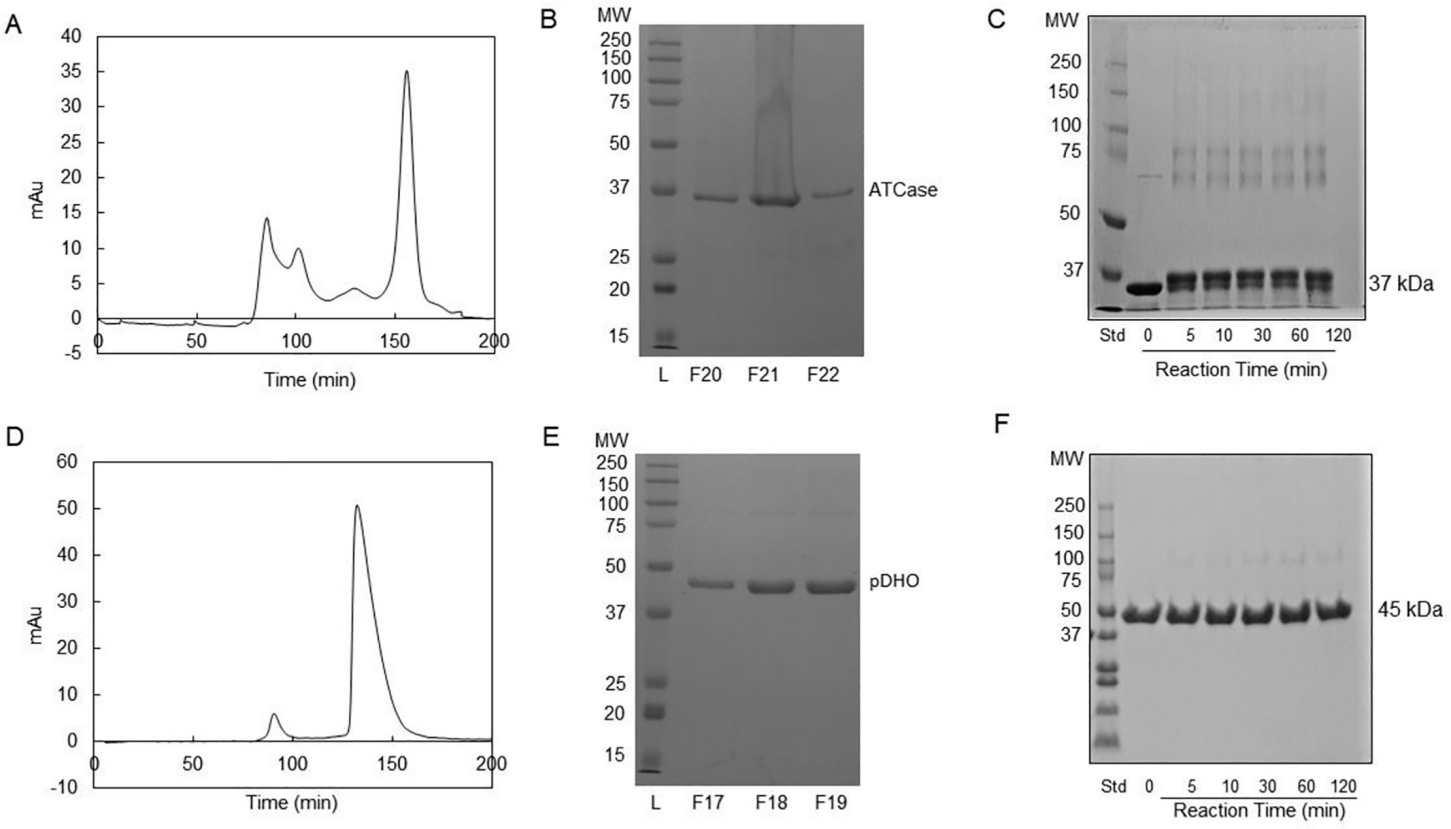
Oligomeric structure of *P*. *aeruginosa* ATCase and pDHO subunits. A) Purified *P*. *aeruginosa* ATCase was subjected to size-exclusion chromatography on a Superdex S-200 Akta column and B) the fractions, F20, F21, and F22, corresponding to the ATCase peak were analyzed by 12% SDS-PAGE. C) Purified recombinant ATCase (45 μM) in 50 mM sodium phosphate, pH 7.5, and 500 mM NaCl, was cross-linked for the indicated times with 5 mM bis(sulfosuccinimidyl)suberate. The reaction was quenched by adding 10 μL of 1 M Tris-HCl, pH 8. Cross-linked species were analyzed by electrophoresis on a 7.5% polyacrylamide gel. D) Purified *P*. *aeruginosa* pDHO was subjected to size-exclusion chromatography. E) Fractions F17, F18, and F19 corresponding to pDHO peak were analyzed by 12% SDS-PAGE and F) chemical crosslinking of pDHO as described for ATCase.

On SDS-PAGE, under denaturing conditions, ATCase had an estimated molecular mass of 36 kDa, in agreement with the mass calculated from the amino acid sequence. ATCase purified by Ni-affinity chromatography was then subjected to size exclusion chromatography on a calibrated Superdex S-200 column (S3 Fig). ATCase eluted as a single species with a molecular mass of 37 kDa indicating that it is monomeric (Fig 2A and 2B). There was no detectable protein in the small shoulder on the leading edge of the peak. The isolated pDHO monomer had a molecular mass of 45 kDa on denaturing SDS gels but eluted as a 90 kDa species on the S-200 size exclusion column indicating that it forms a stable dimer (Fig 2D and 2E).

### Chemical crosslinking

The oligomeric structure of the proteins was also examined by chemical crosslinking. In agreement with the size exclusion chromatography, crosslinking of the ATCase subunit with BS3 (bis(sulfosuccinimidyl) suberate) for up to two hours, showed that the protein remained monomeric (37 kDa) although trace amounts of a higher molecular species were visible (Fig 2C). Although size exclusion chromatography showed that the pDHO is dimeric, no crosslinked species were observed (Fig 2F). This result suggested that the pDHO dimer cannot undergo crosslinking. We are attempting to obtain high quality crystals of *P*. *aeruginosa* ATCase-pDHO but in the meantime comparative modeling was used to examine the interface between the monomers in the putative pDHO dimer. Although there are eight lysines in the pDHO monomer, none are located at or near the dimer interface (S4 Fig) where crosslinking could occur.

### Isolation and composition of the ATCase -pDHO complex

**Fig 3:**
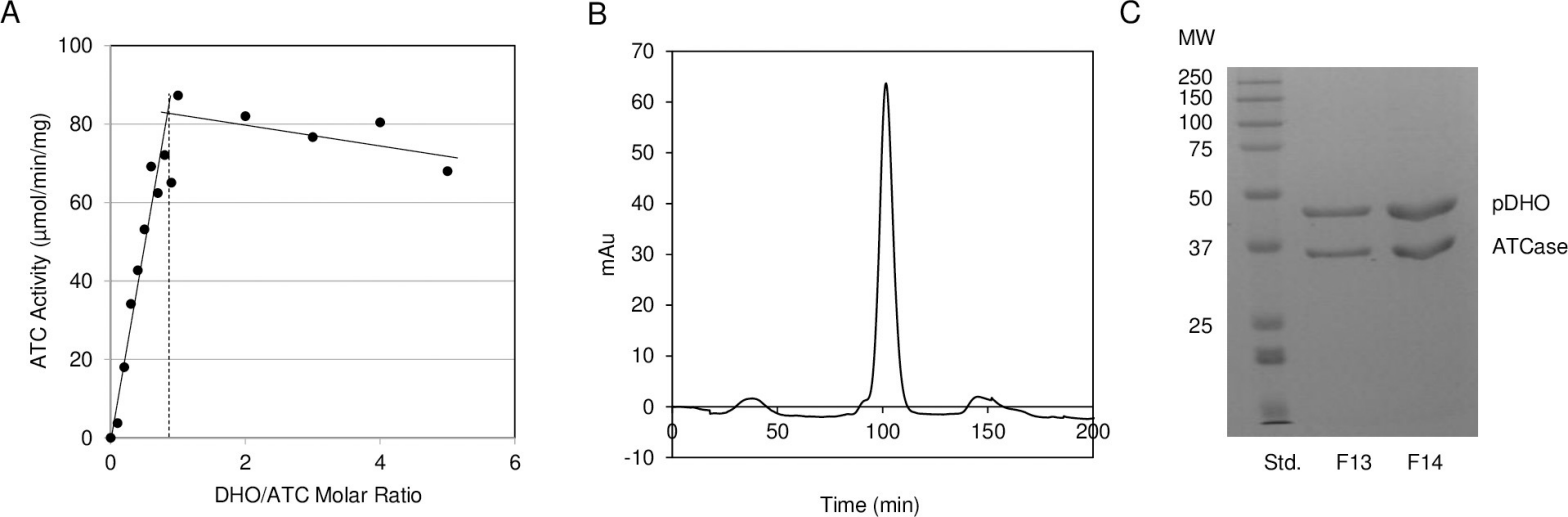
Formation of the ATCase-pDHO complex. A) A fixed constant amount of ATCase (4 μg) was titrated with increasing amounts of pDHO (0–24.3 μg). The ATCase activity was measured at 37°C as described under “Materials and Methods”. B) Purified recombinant ATCase and pDHO were mixed in an equimolar ratio and the complex was analyzed by size-exclusion chromatography on a Sephacryl S-200 column. C) Fractions F13 and F14 corresponding to the complex peak were analyzed on 12% SDS-PAGE.

The isolated ATCase subunit lacked catalytic activity. However, titration of the purified ATCase subunit with pDHO subunit restored the latent catalytic activity (Fig 3A). ATCase activity increased linearly with increasing amounts of pDHO until a molar ratio of ATCase to pDHO was 1:0.97, and the activity then leveled off as pDHO binding site on ATCase became saturated.

The *P*. *aeruginosa* complex was reconstituted by mixing an equimolar ratio of the ATCase and pDHO subunits. The reconstituted complex was then subjected to size exclusion chromatography on a calibrated Superdex S-200 column. The ATCase-pDHO complex eluted in a single peak with a molecular mass of 457 kDa (Fig 3B and S2 Fig). SDS gel electrophoresis confirmed that the complex consisted of equimolar amounts of the two subunits (Fig 3C).

**Fig 4:**
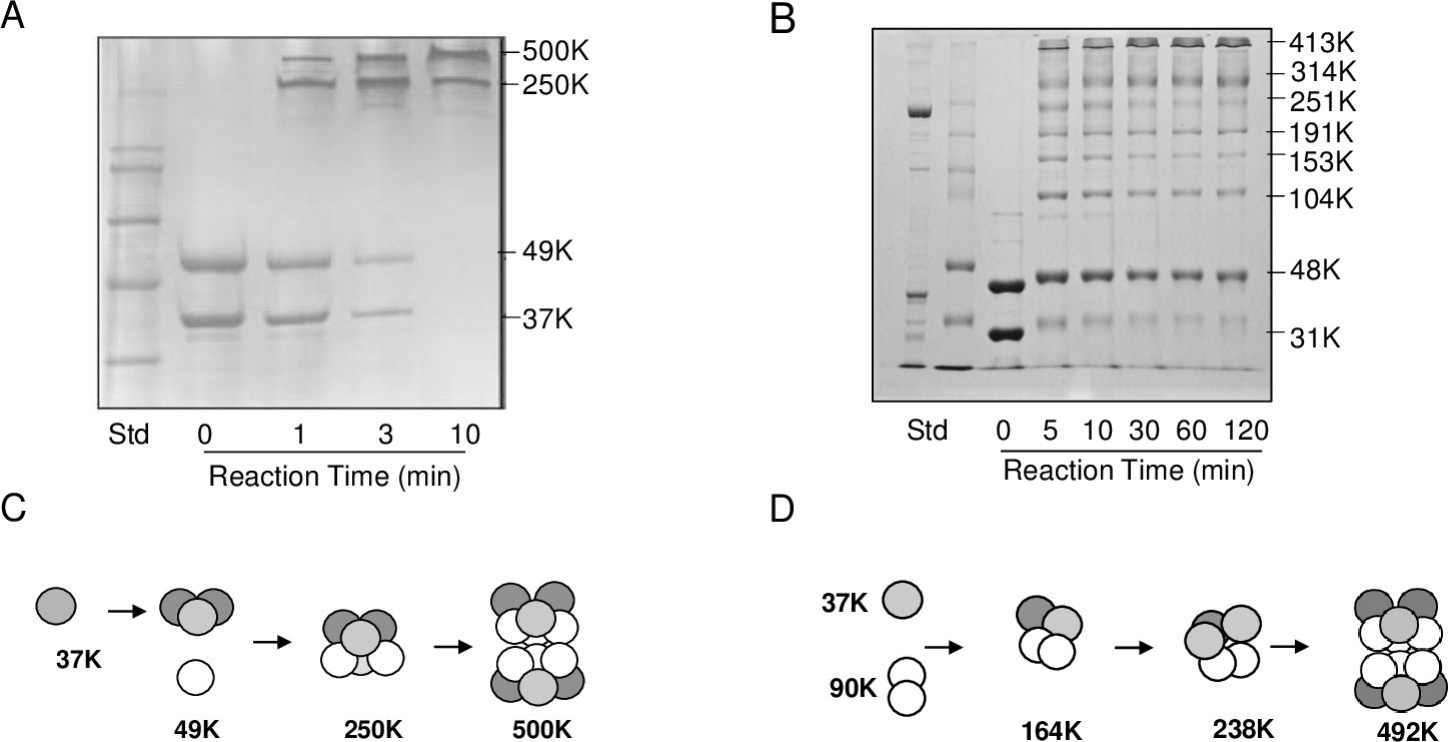
Assembly of the ATCase-pDHO complex. Chemical cross-linking of *A*. *aeolicus* ATCase- DHO with DMS dimethyl suberimidate, Ahuja et al. [22]. C) Purified recombinant *P*. *aeruginosa* ATCase (36 μM) and pDHO (37 μM) in 50 mM sodium phosphate, pH 7.5, and 500 mM NaCl, were cross-linked for the indicated times with 5 mM bis(sulfosuccinimidyl)suberate. The reaction was quenched by adding 10 μl of 1 M Tris-HCl, pH 8. Cross-linked species were analyzed by electrophoresis on a 7.5% polyacrylamide SDS gel. D) The scheme of *P*. *aeruginosa* ATCase-pDHO assembly depicted here is only one of several possible assembly pathways.

Chemical cross-linking of the *A*. *aeolicus* ATCase and DHOase with DMS dimethyl suberimidate gave a limited number of crosslinked species [22] (Fig 4A and 4B). In the absence of the crosslinking reagent, the *P*. *aeruginosa* ATCase-pDHO complex was fully dissociated on the SDS gel into the constituent subunits (lane 3). After the addition of the BS3 (bis(sulfosuccinimidyl) suberate) crosslinker, many more partial species were trapped, suggesting more complex association/dissociation pathway (Fig 4C). An identical profile of crosslinked species was reproducibly obtained in eight crosslinking experiments. The scheme of *P*. *aeruginosa* ATCase-pDHO assembly depicted (Fig 4D) is only one of several possible assembly pathways or there may be no specific pathway with the assembly occurring by the stochastic association of the subunits.

### Steady state kinetics

**Fig 5:**
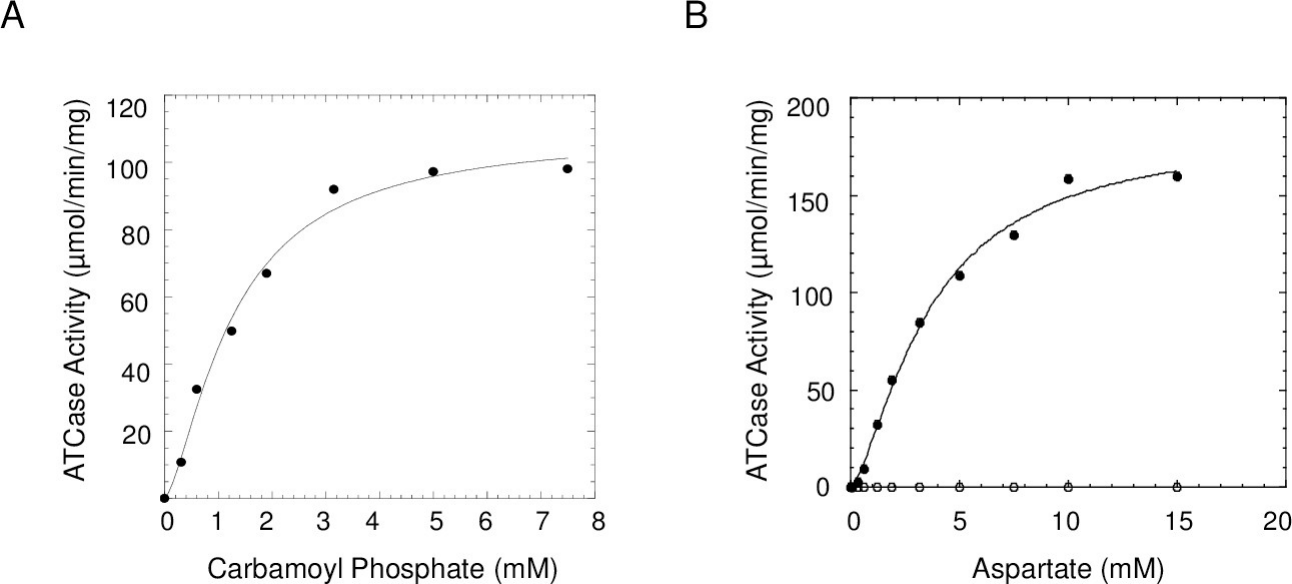
Carbamoyl phosphate and aspartate saturation curves for *P*. *aeruginosa* ATCase kinetics. ATCase activity was measured in the presence (●) of equimolar amounts of pDHO as described under Experimental Procedures, A) Aspartate was fixed at 8 mM and carbamoyl phosphate was the variable substrate. B) Aspartate saturation curves in the presence (●) and absence (○) of pDHO. Carbamoyl phosphate fixed at 10 mM and aspartate as the variable substrate. The saturation curves were fit to the Hill equation: v = V_max_[S]^n^/(K_m_^n^ + S^n^).

The ATCase steady state kinetics parameters were obtained from carbamoyl phosphate (Fig 5A) and aspartate (Fig 5B) saturation curves. As previously reported, *P*. *aeruginosa* ATCase was found to be inactive in the absence of pseudo-DHO. Activity was restored by the addition of an equimolar amount of pDHO to the assay mixture. The carbamoyl phosphate and aspartate saturation curves of *P*. *aeruginosa* ATCase-pDHO complex was slightly sigmoidal. A least square fit of the data to the Hill equation gave a K_m_ for carbamoyl phosphate ([S]_0.5_) of 2.5 mM and a V_max_ of 142 μmoL/min/mg (Table 1). The aspartate saturation curve of ATCase-pDHO had a K_m_ for aspartate of 3.6 mM and a V_max_ of 183 μmoL/min/mg. Both aspartate and carbamoyl phosphate saturation curves exhibit modest sigmoidicity and the Hill coefficients are also given in Table 1. The bi-substrate inhibitor N-phosphonacetyl-L-aspartate is also a potent inhibitor of the of the *P*. *aeruginosa* enzyme (S6 Fig).

**Table 1:** Kinetic parameters of *P*. *aeruginosa* ATCase.

Variable Substrate	Carbamoyl Phosphate (CP)	Aspartate (Asp)
**K**_**m**_ **(mM)**	2.8 ± 0.18	3.6 ± 0.41
**V**_**max**_ **(**μ**mol/min/mg)**	142 ± 7.5	183 ± 4.3
**k**_**cat**_ **(s**^**-1**^**)**	87.6	113.0
**k**_**cat**_**/K**_**m**_ **(s**^**-1**^**M**^**-1**^**)**	3.1 X 10^4^	3.1 X 10^4^
**Hill coefficient**	2.8 ± 0.35	1.4 ± 0.14

#### CD spectroscopy of ATCase, pDHO, and the ATCase-pDHO complex

**Fig 6:**
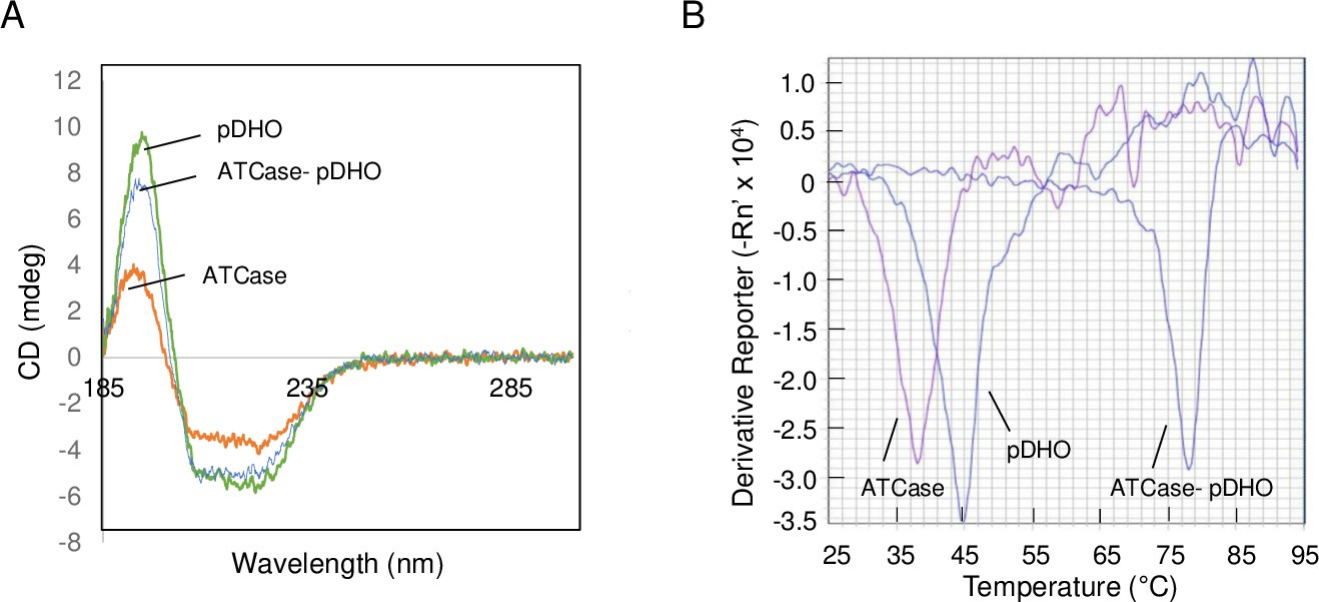
Folding and thermal stability of *P*. *aeruginosa* ATCase, pDHO and the complex. A) The CD spectrum of *P*. *aeruginosa* ATCase, pDHO, and the ATCase-pDHO complex formed by mixing stoichiometric amounts of the two subunits, in 5 mM sodium phosphate, pH 8.0. B) The thermal stability of each purified recombinant protein, ATCase, pDHO, and the ATCase-pDHO complex, was measured by preparing the proteins in buffer containing 50 mM Tris pH 7.5 and 200 mM NaCl, and then subjecting the sample to a thermal shift assay using 500X SYPRO Orange dye. The fluorescence was measured over a temperature range of 25°C to 95°C.

In the absence of pDHO, it is possible that the ATCase trimer does not form because the protein is not folded properly. However, CD spectroscopy of isolated ATCase and pDHO showed that both *P*. *aeruginosa* proteins have at least elements of secondary structure (Fig 6A), (Table 2). The CD spectrum of ATCase-pDHO complex has a similar secondary structure. More subtle changes in the tertiary structure no doubt occur. The secondary structure of BSA was also evaluated by CD spectroscopy as a positive control. Although the relative amount of turns was found to be somewhat higher, the secondary structure contents were similar to those reported previously [32].

**Table 2:** CD spectroscopy.

Secondary Structure*	ATCase	pDHO	ATCase-pDHO	BSA^a^	BSA^b^
	%	%	%	%	%
**Helices**	14 ± 3.3	27 ± 5.6	16 ± 1.8	53	57
**Sheets**	34 ± 2.6	23 ± 5.8	33 ± 1.0	4	6
**Turns**	22 ± 0.3	27 ± 5.6	22 ± 0.2	32	14
**Undetermined**	30 ± 0.9	28 ± 0.8	27 ± 2.5	22	24

*The average of three determinations and standard deviations.

^a^Determined in this study

^b^Literature value [32]

### Thermal stability of ATCase, pDHO, and the ATCase-pDHO complex

A thermal shift assay was employed to determine the relative stability of the isolated subunits and the complex (Fig 6B). The melting temperature of the proteins was analyzed by calculating the first derivative of the fluorescence vs. temperature plot using the Applied Biosystems 7500 software. ATCase and pDHO subunits had melting temperatures of 38°C and 44°C, respectively. When the reconstituted ATCase-pDHO complex was subjected to thermal shift, there was a dramatic positive shift in the melting temperature of the complex to 77°C indicating that while the ATCase and pDHO subunits are relatively unstable, the ATCase-pDHO dodecamer is significantly more stable.

### Activation of ATCase

The possibility was considered that there may be critical regions of the protein that are intrinsically disordered. The Protein Disorder Prediction System (PrDOS) was employed to identify these disordered regions. This analysis showed that the first 11 residues on the amino end and the last 13 residues on the carboxyl end of the ATCase subunit are disordered. These disordered regions are not present in the shorter *E*. *coli* ATCase polypeptide. The inactivity of ATCase could be explained if the disordered extensions interfere with ATCase trimer formation in the absence of the pDHO, a pre-requisite for the enzyme’s activity. The *pyr*B gene of *P*. *aeruginosa* ATCase without the first 11 residues (delN ATCase) and without both the first 11 and last 13 residues (delNC ATCase) were cloned into T7 pETite vector and purified as described for the native subunit.

The overexpression of mutant ATCases resulted in formation of insoluble inclusion bodies, therefore, mutant ATCases could not be tested for activity in the absence of pDHO. However, when the constructs were co- transformed and co-purified with *pyr*X gene encoding pDHO, mutant ATCases in complex with pDHO were found to be active suggesting that the truncation of the first 11 or last 13 residues did not affect the formation of ATCase-pDHO complex. Furthermore, ATCase was subjected to controlled proteolysis by elastase. The rationale for this experiment was that the elastase would cleave accessible and disordered regions of the protein. If these regions exert any inhibitory constraints, the ATCase may be activated. However, the time course of controlled proteolysis did not induce recovery of the latent ATCase activity.

### Nucleotide inhibition

**Fig 7:**
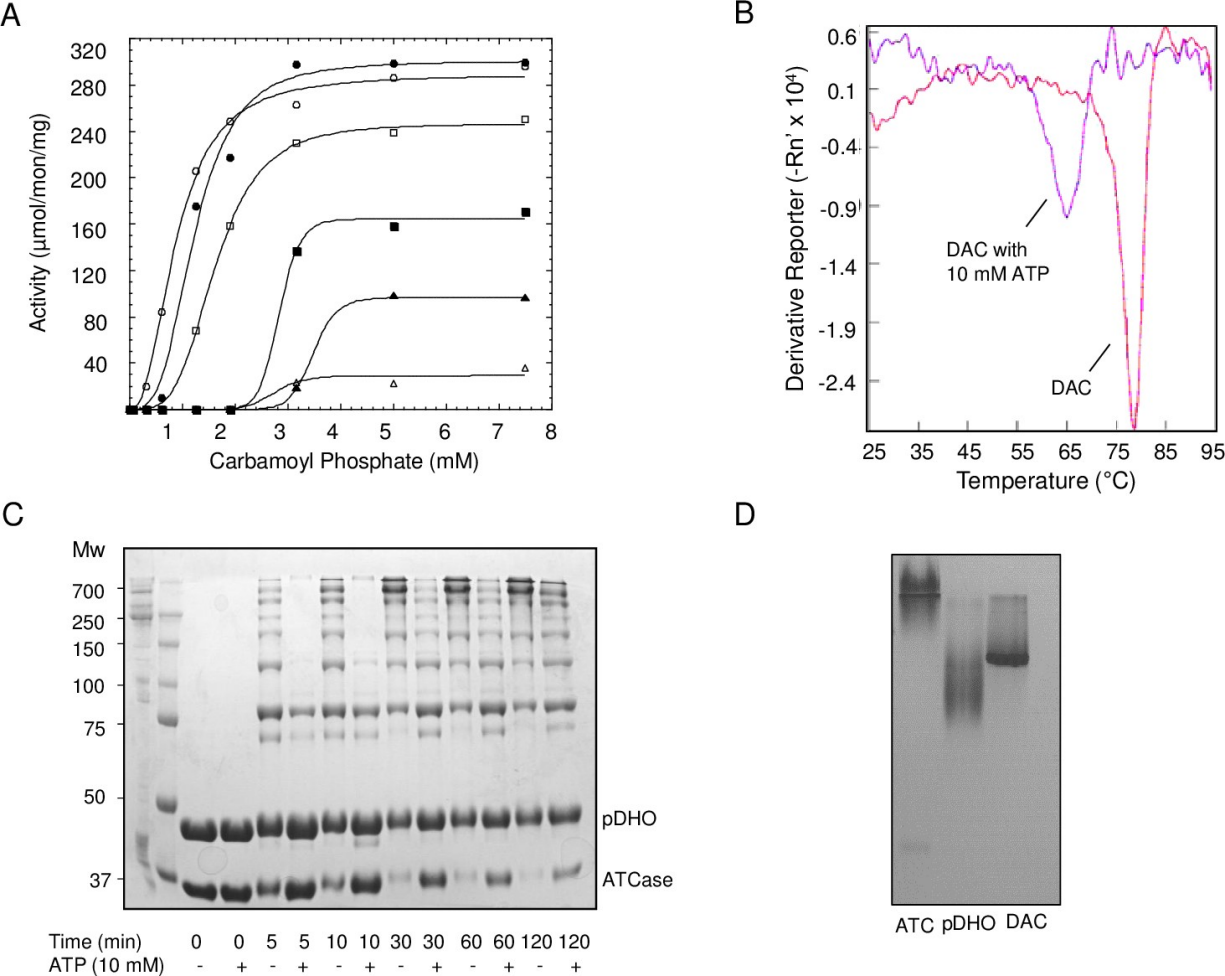
Nucleotide inhibition. The ATCase activity was measured in the presence of equimolar pDHO as described above. A) The activity of the ATCase-pDHO complex was measured with carbamoyl phosphate as the variable substrate, aspartate fixed at 8 mM in the absence of ATP (○) and in the presence of 0.5 mM (●), 1 mM (□), 2.5 mM (■), 5 mM (▲), and 10 mM (Δ) ATP. B) The preformed ATCase-pDHO complex prepared in buffer containing 50 mM Tris pH 7.5 and 200 mM NaCl was subjected to thermal shift assay using 500X SYPRO Orange dye, in the presence and absence of 10 mM ATP. The fluorescence was measured over a temperature range of 25°C to 95°C. C) Purified recombinant ATCase (36 μM) and pDHO (37 μM) in 50mM sodium phosphate, pH 7.5, and 500 mM NaCl, were cross-linked for the indicated times with 5 mM bis(sulfosuccinimidyl)-suberate in the absence and presence of 10 mM ATP, as described in the Experimental Procedures. D) Gel-filtered ATCase, pDHO, and the ATC-pDHO complex were preincubated with 10 mM ATP and subjected to native gel electrophoresis with 10 mM ATP in the gel matrix and running buffer.

As previously demonstrated, the ATCase-pDHO complex is strongly inhibited by nucleotide triphosphates (Fig 7A). Carbamoyl phosphate saturation curves showed mixed inhibition, decreasing k_cat_, and to a lesser extent increasing K_m_ (Table 3). The most striking effect is a large increase in the Hill coefficient as the ATP concentration increases. When ATCase-pDHO complex was cross-linked with BS3 in the presence of 10 mM ATP, (Fig 7C) the complex appeared to be less stable as indicated by the appearance of fewer and reduced levels of intermediate species. Additionally, thermal shift data showed a negative 13°C shift of the melting temperature of the ATCase-pDHO complex in the presence of ATP—a further indication that ATP destabilizes the ATCase-pDHO complex (Fig 7B). Furthermore, the ATCase-pDHO complex was preincubated with 10 mM ATP and analyzed on 7.5% native gel with 10 mM ATP in the gel matrix and gel running buffer. The ATCase-pDHO complex ran as a single band on native gel containing 10 mM ATP indicating that the complex, although destabilized is not completely dissociated by ATP (Fig 7D). Moreover, the mutations in ATCase, delN or delNC, did not affect ATP inhibition suggesting that the ATP binding site is not present within the first 11 residues of N terminal as previously suggested.

**Table 3:** ATP inhibition.

ATP	V_max_	K_m_	n_H_
mM	μmol/min/mg	mM	
0	289 ± 5.0	0.88 ± 0.03	2.4
0.5	300 ± 10	1.22 ± 0.07	3.2
1.0	247 ± 2.7	1.62 ± 0.02	3.8
2.5	164 ± 2.6	2.83 ± 0.32	14.7
5	97 ± 1.1	3.53 ± 0.10	12.7
10	29 ± 2.7	2.76 ± 0.48	10.3

## Discussion

Previous studies have shown that *Pseudomonas* ATCases have some unusual properties. All ATCase catalytic subunits from bacteria, fungi, animals and plants [1–10, 33–35] have homologous sequences, have the same catalytic residues and mechanism and form stable catalytically active trimers. Although Schurr et al. [26] did not isolate the ATCase subunit they showed the ATCase activity in *Pseudomonas putida* was dependent on the presence of the pDHO domain and correctly concluded that it was necessary for the stability of the ATCase-pDHO complex. In this study we have cloned and expressed both the *P*. *aeruginosa* inactive ATCase and pDHO subunits and found that a relatively stable dodecameric complex with full ATCase activity could be reconstituted when the two subunits were mixed in a 1:1 stoichiometric molar ratio.

Unlike all other ATCase that have been previously studied, the ATCase chain from *P*. *aeruginosa* is monomeric and does not assemble into a catalytically active trimer unless the pDHO subunit is present. No significant amount of the ATCase trimer could be detected by size exclusion chromatography or by chemical crosslinking. In contrast, chromatography indicated that the pDHO subunit formed a stable dimer, although the dimer did not crosslink presumably because there are no appropriately positioned lysine residues at the dimer interface. The failure to form a stable ATCase trimer accounts for its lack of catalytic activity since the active site of every known ATCase requires a specific Ser and a Lys residue from the adjacent subunit in the trimer for activity.

The three very important interactions between *E*. *coli* ATCase monomers in the catalytic trimer have been identified to be Lys-41, Asp-100, and Asp-90 of one monomer that form salt links with Glu-37, Arg-65, and Arg-269 of the adjacent subunit [36, 37]. Multiple sequence alignment of *P*. *aeruginosa* ATCase with other trimeric ATCases (S1 Fig) showed that two of the three interactions are partially conserved in *P*. *aeruginosa*. However, understanding why the stable trimer cannot form in the absence pDHO must await the three-dimensional structure of the dodecameric complex, now in progress.

CD analysis showed that both the isolated subunits can spontaneously assume a typical secondary structure, although neither ATCase nor pDHO is very stable with melting temperatures as determined by the thermal shift assay were 38°C and 44°C respectively. Formation of the complex appreciably stabilizes the protein raising the melting temperature to 77°C. Time-dependent chemical crosslinking of the reconstituted dodecamer of the *A*. *aeolicus* ATCase-DHOase complex showed a progressive disappearance of the monomers and the formation of the 240 kDa hexamer which gradually disappears with the formation of the 480 kDa dodecamer. In contrast, crosslinking of preformed *P*. *aeruginosa* ATCase-pDHO gave a complex but highly reproducible pattern of 8 bands, that gradually gave way to species of higher molecular mass. This pattern may have resulted from the intrinsic instability of the subunits and the lack of pDHO crosslinking. Each ATCase monomer interacts with both other monomers in the trimer and with two different sites on the pDHO, while each pDHO interacts with two different ATCase monomers and two other pDHO subunits so these interactions would be expected give rise to a large number of different intermediate species. Similarly, the assembly of the dodecamer is likely to be complex with the scheme shown (Fig 4D) being only one of many possible pathways or there may be no specific pathway with the assembly occurring by the stochastic association of the subunits.

As reported previously [24] micromolar levels of nucleotide tri- and di-phosphates competitively inhibit *P*. *fluorescens* when carbamoyl phosphate is limiting (7.5–30 μM). At higher carbamoyl phosphate concentrations (10 mM), nucleotides are non-competitive inhibitors [38]. The regulatory logic of these phenomena is elusive. *E*. *coli* ATCase is allosterically inhibited by UTP and CTP and activated by ATP. The rationale is that this regulatory mechanism provides a balance between t synthesis of pyrimidines and purines. However, *P*. *aeruginosa* ATCase is inhibited by both pyrimidines and purines. The inhibitors tend to shift the saturation curve to higher substrate concentrations and increase cooperative substrate binding and Hill coefficient. The Monod, Wyman and Changeux model stipulates that the upper limit of the Hill coefficient equals the number of substrate binding sites. The Hill coefficients for *P*. *aeruginosa* ATCase here and in a previous study [27] far exceed six, (Table 3, n = 13–14) the number of effector binding sites suggesting that the observed sigmoidicity does not reflect true homotropic or cooperative substrate binding. Rather the sigmoidicity of these plots probably results from the weakening of the ATCase -pDHO intersection’s and accompanying inactivation of the ATCase trimer. The ATP binding site was previously shown [25] to be located on the ATCase catalytic subunit by affinity labeling with the radiolabeled ATP analog, [^3^H]-FSBA. Previous studies [26] provided evidence that ATP binds to the 11-residue extension on the amino end of the catalytic polypeptide. However, subcloning of deletion mutants lacking the extension on the amino end and the carboxyl ends of the protein were inhibited by ATP. We conclude that the nucleotide inhibition is a consequence of the destabilization of the ATCase-pDHO complex and does not represent an allosteric regulatory mechanism. How then is pyrimidine biosynthesis regulated in *P*. *aeruginosa*? A possible explanation is that carbamoyl phosphate synthetase (CPSase) which provides the ATCase substrate, carbamoyl phosphate regulates ATCase. *P*. *aeruginosa* CPSase is controlled by metabolites in the arginine biosynthetic pathway but is also feedback inhibited by CTP [39].

In summary, although the isolated ATCase from *P*. *aeruginosa* has a secondary structure, it is relatively unstable and does not self-associate to form a stable trimer. Association with the pDHO subunit is therefore necessary to stabilize the catalytically active timer. ATP induced destabilization of the ATCase-pDHO complex probably results in the inactivation of the ATCase catalytic subunit.

## Supporting information

S1 Fig Sequence similarity of ATCases. A CLUSTAL O (1.2.4) multiple sequence alignment of ATCase catalytic chain or domain of ATCase from *E*. *coli* (Ec), *Homo sapiens* (Hs), *Aquifex aeolicus* (Aa), *Pseudomonas aeruginosa* (Pa) and *Staphylococcus aureus* (Sa). Active site residues are highlighted in red; H5 and H12 represent the residues that connect the carbamoyl phosphate and aspartate domains of ATCase; residues involved in monomer-monomer interactions are boxed in red; amino and carboxyl end extensions of *P*. *aeruginosa* ATCase are highlighted in green.
(DOCX)

S2 Fig Sequence of the constructs. The *P*. *aeruginosa* proteins were expressed in *E*. *coli* using the Lucigen Expresso^™^ T7 Cloning and Expression System. The vector is diagramed on the right https://www.lucigen.com/docs/manuals/MA101-Expresso-T7-Cloning-&-Expression-System.pdf The vector appends six histidines to the amino end and has a stop codon on the carboxyl end.
(DOCX)

S3 Fig Calibration of the size exclusion column. A. The Superdex S-200 column was calibrated with four proteins of known molecular mass; thyroglobulin (670 kDa) eluted at 43.35 ml. (90.70 ml), γ-globulin 158 kDa) eluted at 59.57 ml (119.13 ml), (ovalbumin (44 kDa) eluted at 75.54 ml (151.07 min), myoglobulin eluted at 86.97 (75.54 ml 59.57 ml (173.94 ml). Vitamin B12, 1.35 kDa which eluted at 104.59 ml (209.17 min) defines the total volume of the column. B. A plot of the log of the molecular mass versus the elution times. The arrows indicate the elution times of the *P*. *aeruginosa* proteins.
(DOCX)

S4 Fig Model of the DHO dimer. A homology model of the pDHO dimer calculated using SWISS-MODEL (ExPAYs Bioinformatics Research Portal). *A*. *aeolicus* ATCase-DHOase was used as the template. The lysine residues are displayed in ball and stick format and are colored red.
(DOCX)

S5 Fig Kinetic data. Data for the carbamoyl phosphate, aspartate saturation curves and the inhibition by PALA.
(DOCX)

S6 Fig PALA saturation curves. The inhibition of *P*. *aeruginosa* ATCase inhibition by PALA. Carbamoyl phosphate saturation curves of *P*. *aeruginosa* ATCase in the presence of 0 (●), 1 nM (○), 25 nM (■), 100 nM (□), 500 nM (X) of PALA was carried out as described in the text. The complex was formed by mixing stoichiometric concentrations of ATCase (6 μg) and pDHO (7.5 ug). The assay was conducted for 2 min at 37^○^ and 8 mM aspartate. The curves were fit using the program KaleidaGraph (Synergy Software) to the Hill equation: v = (S)^n^V_max_/(K_m_^n^+S^n^) where n is the Hill coefficient.
(DOCX)
