# Supplementary material for: Characterization and assembly of the Pseudomonas aeruginosa aspartate transcarbamoylase-pseudo dihydroorotase complex
Source: PLoS One. 2020 Mar 3;15(3):e0229494. doi: 10.1371/journal.pone.0229494 (PMC7053772; doi:10.1371/journal.pone.0229494)
Supplement: S1 Fig — A CLUSTAL O (1.2.4) multiple sequence alignment of ATCase catalytic chain or domain of ATCase from E. coli (Ec), Homo sapiens (Hs), Aquifex aeolicus (Aa), Pseudomonas aeruginosa (Pa) and Staphylococcus aureus (Sa). Active site residues are highlighted in red; H5 and H12 represent the residues that connect the carbamoyl phosphate and aspartate domains of ATCase; residues involved in monomer-monomer interactions are boxed in red; amino and carboxyl end extensions of P. aeruginosa ATCase are highlighted in green. (DOCX) [file pone.0229494.s001.docx]

**S1 Fig. Alignment of the catalytic subunit or domain of aspartate transcarbamoylase**

Ec 1Q95_C -----------ANPLYQKHIISINDLSRDDLNLVLATAAKL-----KANPQPELLKHKVI 44

Hs 5G1P_D ---GPMS--PLLHSLVGQHILSVQQFTKDQMSHLFNVAHTLRM-MVQKERSLDILKGKVM 54

Aa 3D6N_B ----------------MRSLISSLDLTREEVEEILKYAKEFKEGKEETIKA-------SA 37

Pa AAA25976.1 MPTDAKRPLQLNDQGQLRHFISLDGLPRELLTEILDTADSFLEVGARAVKKVPLLRGKTV 60

Sa BAF78073.1 ----------------MNHLLSMEHLSTDQIYKLIQKASQFK----SGERQLPNFEGKYV 40

. ::* : : : :: * :

1Q95_C ASCFF**E**ASTRTRLSF**E**TSMHRLGAS**V**VGFSDSANT**S**LGKKGETLA**D**TISVIST-YV**D**AIV 103

5G1P_D ASMFYEVSTRTSSSFAAAMARLGGAVLSFSEAT--SSVQKGESLADSVQTMSC-YADVVV 111

3D6N_B VL**F**FSEPSTRTR**L**SFEKAAREL**G**IETYLVSGS**E**--SSTVKGESF**F**DTLKTFEGL**G**FDYVV 95

AAA25976.1 CNVFFENSTRTRTTFELAAQRLSADVISLNVST--SSTSKGETLTDTLRNLEAMAADMFV 118

BAF78073.1 ANLFFENSTRTKCSFEMAELKLGLKTISFETST--SSVSKGESLYDTCKTLESIGCDLLV 98

* * **** :* : .*. . .. : * ***:: *: :. * .*

H5

1Q95_C MRHPQE**G**AARLATE-FS**G**NVPVLNAGD**G**SNQHPTQTLLDLFTIQETQGRLDNLHVAM**V**GD 162

5G1P_D LRHPQPGAVELAAK-H-CRRPVINAGDGVGEHPTQALLDIFTIREELGTVNGMTITMVGD 169

3D6N_B FRVPFVFF-PYKEIVKSLNLRLVNAGDGTHQHPSQGLIDFFTIKEHFGEVKDLRVLYVGD 154

AAA25976.1 VRHSDSGAAHFIAEHVSPNVAVINGGDGRHAHPTQGMLDMLTIRRHKGNFEQLSVAIVGD 178

BAF78073.1 IRHPFNN---YYEKLANINIPIANAGDGSGQHPTQSLLDLMTIYEEYGYFEGLNVLICGD 155

.* . : *.*** **:* ::*::** . * .. : : **

1Q95_C LKYGRTV**H**SLTQALAKF**D**GNRFYFIAPDALAMPQYILDMLDEKGIAWSLHSSIEEVM**A**EV 222

5G1P_D LKHGRTVHSLACLLTQYRVS-LRYVAPPSLRMPPTVRAFVASRGTKQEEFESIEEALPDT 228

3D6N_B IKHSRVFRSGAPLLNMFGAKI-GVCGPKTLI-P-----RDVE-VFKVDVFDDV**D**KGIDWA 206

AAA25976.1 ILHSRVARSNMLALKTLGCPDIRVIAPATLL-P-----IGLEEQYGVRVFTNADEGLKDV 232

BAF78073.1 IKNSRVARSNYHSLKALGANVMF-NSPNAWI-D-----DSLE-----APYVNIDDVIETV 203

: .*. :* * .* : . . . :. : .

1Q95_C DILYMTR**V**QKERLDP-SE**Y**AN----VKAQFVL**R**ASDLHNAKA**N**MKVLHPLP--R**V**DEIAT 275

5G1P_D DVLYMTRIQKERFGSTQEYEA----CFGQFILTPHIMTRAKKKMVVMHPMP--RVNEISV 282

3D6N_B DVV**I**WLRLQKERQKE--NYIPSESSYFKQFGLTKERFEKV---KLYMH**P**GPVNRNVDI**D**H 261

AAA25976.1 DVVIMLRLQRERMQG--GLLPSEGEFFKLYGLTEKRLKLAKPDAIVMHPGPINRGVEIES 290

BAF78073.1 DIVMLLRIQHERHGLAEETRFAADDYHQKHGLNEVRYNKLQEHAIVMHPAPVNRGVEIQS 263

H12

*:: *:*:** . * :** * * :*

1Q95_C DVDK**T**PHAWYFQQA**G**NGIFARQALLALVLNRDLVL--------- 310

5G1P_D EVDSDPRAAYFRQAENGMYIRMALLATVLGRF------------ 314

3D6N_B ELV**Y**TEKS**L**IQEQVKNGIPVRKAIYKFLWT-------------- 291

AAA25976.1 AVADGAQSVILNQVTYGIAIRMAVLSMAMSGQNTQRQLEQEDAE 334

BAF78073.1 DLVEASKSRIFKQMENGVYLRMAVIDELLK-------------- 293

: :: .* *: * *:

Ec 1Q95_C -----------ANPLYQKHIISINDLSRDDLNLVLATAAKL-----KANPQPELLKHKVI 44

Hs 5G1P_D ---GPMS--PLLHSLVGQHILSVQQFTKDQMSHLFNVAHTLRM-MVQKERSLDILKGKVM 54

Aa 3D6N_B ----------------MRSLISSLDLTREEVEEILKYAKEFKEGKEETIKA-------SA 37

Pa AAA25976.1 MPTDAKRPLQLNDQGQLRHFISLDGLPRELLTEILDTADSFLEVGARAVKKVPLLRGKTV 60

Sa BAF78073.1 ----------------MNHLLSMEHLSTDQIYKLIQKASQFK----SGERQLPNFEGKYV 40

. ::* : : : :: * :

1Q95_C ASCFF**E**ASTRTRLSF**E**TSMHRLGAS**V**VGFSDSANT**S**LGKKGETLA**D**TISVIST-YV**D**AIV 103

5G1P_D ASMFYEVSTRTSSSFAAAMARLGGAVLSFSEAT--SSVQKGESLADSVQTMSC-YADVVV 111

3D6N_B VL**F**FSEPSTRTR**L**SFEKAAREL**G**IETYLVSGS**E**--SSTVKGESF**F**DTLKTFEGL**G**FDYVV 95

AAA25976.1 CNVFFENSTRTRTTFELAAQRLSADVISLNVST--SSTSKGETLTDTLRNLEAMAADMFV 118

BAF78073.1 ANLFFENSTRTKCSFEMAELKLGLKTISFETST--SSVSKGESLYDTCKTLESIGCDLLV 98

* * **** :* : .*. . .. : * ***:: *: :. * .*

H5

1Q95_C MRHPQE**G**AARLATE-FS**G**NVPVLNAGD**G**SNQHPTQTLLDLFTIQETQGRLDNLHVAM**V**GD 162

5G1P_D LRHPQPGAVELAAK-H-CRRPVINAGDGVGEHPTQALLDIFTIREELGTVNGMTITMVGD 169

3D6N_B FRVPFVFF-PYKEIVKSLNLRLVNAGDGTHQHPSQGLIDFFTIKEHFGEVKDLRVLYVGD 154

AAA25976.1 VRHSDSGAAHFIAEHVSPNVAVINGGDGRHAHPTQGMLDMLTIRRHKGNFEQLSVAIVGD 178

BAF78073.1 IRHPFNN---YYEKLANINIPIANAGDGSGQHPTQSLLDLMTIYEEYGYFEGLNVLICGD 155

.* . : *.*** **:* ::*::** . * .. : : **

1Q95_C LKYGRTV**H**SLTQALAKF**D**GNRFYFIAPDALAMPQYILDMLDEKGIAWSLHSSIEEVM**A**EV 222

5G1P_D LKHGRTVHSLACLLTQYRVS-LRYVAPPSLRMPPTVRAFVASRGTKQEEFESIEEALPDT 228

3D6N_B IKHSRVFRSGAPLLNMFGAKI-GVCGPKTLI-P-----RDVE-VFKVDVFDDV**D**KGIDWA 206

AAA25976.1 ILHSRVARSNMLALKTLGCPDIRVIAPATLL-P-----IGLEEQYGVRVFTNADEGLKDV 232

BAF78073.1 IKNSRVARSNYHSLKALGANVMF-NSPNAWI-D-----DSLE-----APYVNIDDVIETV 203

: .*. :* * .* : . . . :. : .

1Q95_C DILYMTR**V**QKERLDP-SE**Y**AN----VKAQFVL**R**ASDLHNAKA**N**MKVLHPLP--R**V**DEIAT 275

5G1P_D DVLYMTRIQKERFGSTQEYEA----CFGQFILTPHIMTRAKKKMVVMHPMP--RVNEISV 282

3D6N_B DVV**I**WLRLQKERQKE--NYIPSESSYFKQFGLTKERFEKV---KLYMH**P**GPVNRNVDI**D**H 261

AAA25976.1 DVVIMLRLQRERMQG--GLLPSEGEFFKLYGLTEKRLKLAKPDAIVMHPGPINRGVEIES 290

BAF78073.1 DIVMLLRIQHERHGLAEETRFAADDYHQKHGLNEVRYNKLQEHAIVMHPAPVNRGVEIQS 263

H12

*:: *:*:** . * :** * * :*

1Q95_C DVDK**T**PHAWYFQQA**G**NGIFARQALLALVLNRDLVL--------- 310

5G1P_D EVDSDPRAAYFRQAENGMYIRMALLATVLGRF------------ 314

3D6N_B ELV**Y**TEKS**L**IQEQVKNGIPVRKAIYKFLWT-------------- 291

AAA25976.1 AVADGAQSVILNQVTYGIAIRMAVLSMAMSGQNTQRQLEQEDAE 334

BAF78073.1 DLVEASKSRIFKQMENGVYLRMAVIDELLK-------------- 293

: :: .* *: * *:
