# Supplementary material for: Characterization and assembly of the Pseudomonas aeruginosa aspartate transcarbamoylase-pseudo dihydroorotase complex
Source: PLoS One. 2020 Mar 3;15(3):e0229494. doi: 10.1371/journal.pone.0229494 (PMC7053772; doi:10.1371/journal.pone.0229494)
Supplement: S2 Fig — The P. aeruginosa proteins were expressed in E. coli using the Lucigen Expresso™ T7 Cloning and Expression System. The vector is diagramed on the right https://www.lucigen.com/docs/manuals/MA101-Expresso-T7-Cloning-&-Expression-System.pdf The vector appends six histidines to the amino end and has a stop codon on the carboxyl end. (DOCX) [file pone.0229494.s002.docx]

**S2 Fig.** **Sequence of the Expression Constructs**

**
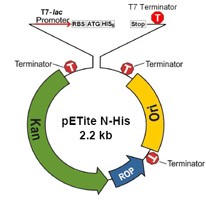
A. The Lucigen Expresso™ T7 Cloning and Expression System**.

**B. *Pseudomonas aeruginosa* aspartate transcarbamoylase pyB coding sequence**

atg cac cac cac cac cac cac

1 atgccgacag acgccaagcg cccgctgcag ctcaacgacc agggccagct gcgccacttc

61 atctcgctcg acggattgcc ccgcgagctg ctcaccgaaa tcctcgatac cgccgattcc

121 ttcctggagg tcggcgcccg cgcggtgaaa aaggtcccgc tgctgcgcgg caagaccgtc

………. 660 nucleotides……..

781 ggcctgaccg agaagcgcct gaagctggcc aagccggatg ccatcgtcat gcaccctggc

841 ccgatcaacc gtggcgtgga gatcgagtcg gcggtggccg acggggccca gtcggtgatc

901 ctcaaccagg tcacctacgg catcgccatc cgcatggcgg tgctgtccat ggccatgagc

961 ggccagaaca cccaacgcca gctggaacag gaggacgccg a

**C.  *Pseudomonas aeruginosa* pseudo-dihydroorotase, pyrX, coding sequence**

atg cac cac cac cac cac cac

1002 ccatc agtatccgag

1021 gcgcccgcgt catcgacccg gccagtgacc tggaccaggt cggcgacctc cacatcgagg

1081 ctggcaagat cgtcgccatc ggcgccgcgc cggccggctt cagcgcccag aagaccctcg

1141 acggcgccgg cctggtggcc gccgcgggac tggtcgatct gagcgtcgcc ctgcgcgagc

1201 cgggctatgg acgcaagggc aacgtcgaaa gcgagacccg cgccgccgcg gccggcggca

…………… 900 nucleotides………..

2101 ggcgcctggc ggtgggccag gccgccgacc tggtgctgtt cgacccgcag ggttcgaccc

2161 tggccggtga gagctggtac tcgaaggggc agaacagccc gttcgtcggt cactgcctgc

2221 cgggacgagt gcgctacacc ctggtggacg gacacctgac ccacgagggc tga
