# Supplementary material for: Characterization and assembly of the Pseudomonas aeruginosa aspartate transcarbamoylase-pseudo dihydroorotase complex
Source: PLoS One. 2020 Mar 3;15(3):e0229494. doi: 10.1371/journal.pone.0229494 (PMC7053772; doi:10.1371/journal.pone.0229494)
Supplement: S3 Fig — A. The Superdex S-200 column was calibrated with four proteins of known molecular mass; thyroglobulin (670 kDa) eluted at 43.35 ml. (90.70 ml), γ-globulin 158 kDa) eluted at 59.57 ml (119.13 ml), (ovalbumin (44 kDa) eluted at 75.54 ml (151.07 min), myoglobulin eluted at 86.97 (75.54 ml 59.57 ml (173.94 ml). Vitamin B12, 1.35 kDa which eluted at 104.59 ml (209.17 min) defines the total volume of the column. B. A plot of the log of the molecular mass versus the elution times. The arrows indicate the elution times of the P. aeruginosa proteins. (DOCX) [file pone.0229494.s003.docx]

**S3 Fig. Calibration of the Superdex S-200 Column**

B

Log (Mr)

Time (min)

ATC

pDHO

ATC-pDHO


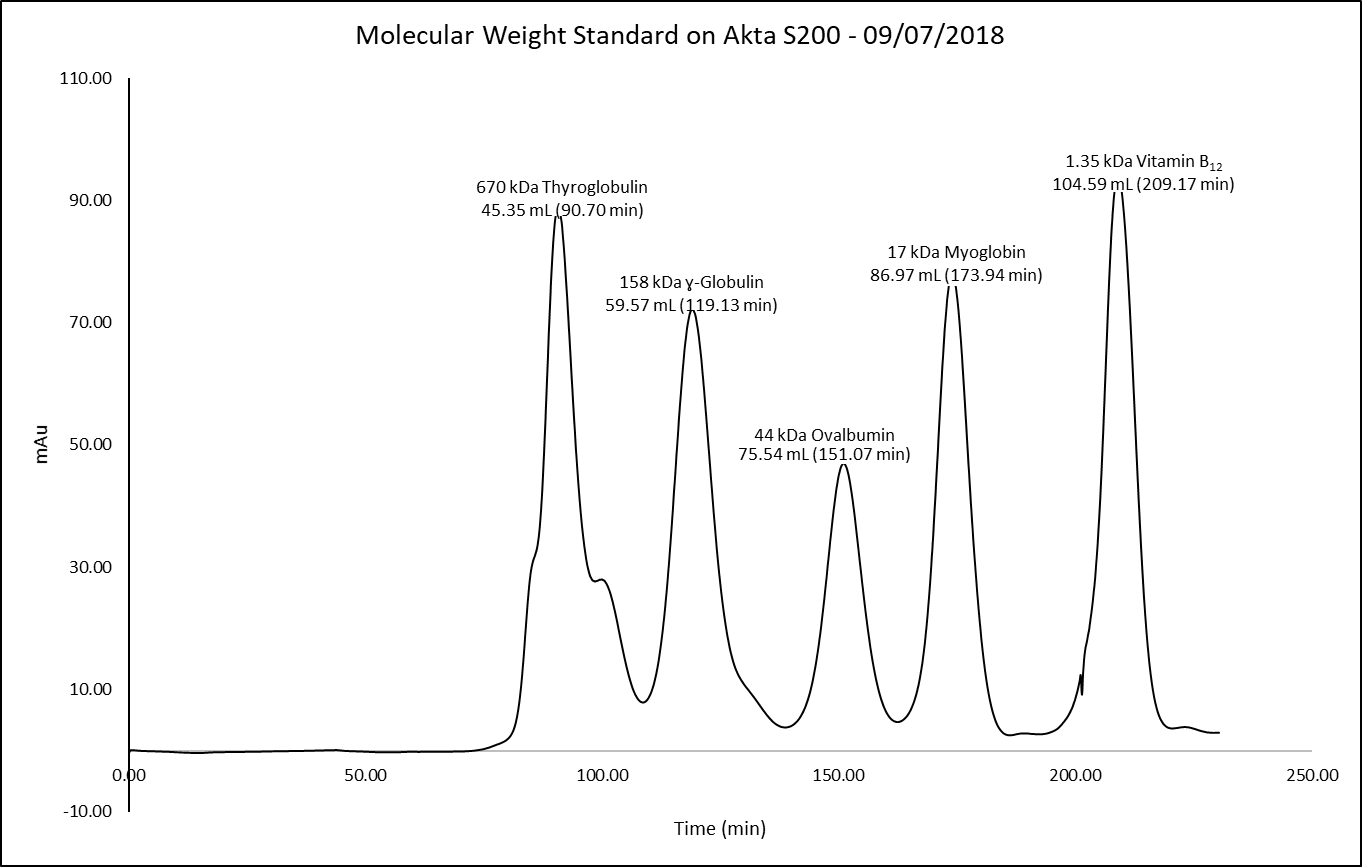


γ-Globulin

Ovalbumin

Myoglobulin

Vitamin B_12_

Thyroglobulin

90

700

50

30

10

A

100 150 200
 Time (min)
