# Supplementary figures and images for: Characterization and assembly of the Pseudomonas aeruginosa aspartate transcarbamoylase-pseudo dihydroorotase complex
Source: PLoS One. 2020 Mar 3;15(3):e0229494. doi: 10.1371/journal.pone.0229494 (PMC7053772; doi:10.1371/journal.pone.0229494)

S4 Fig. Model of the DHO Dimer


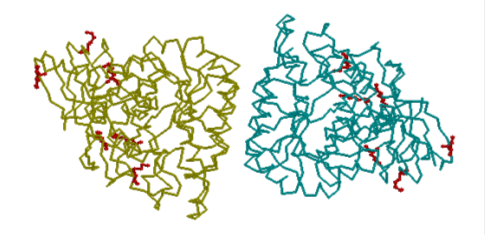

Supplement: S4 Fig — A homology model of the pDHO dimer calculated using SWISS-MODEL (ExPAYs Bioinformatics Research Portal). A. aeolicus ATCase-DHOase was used as the template. The lysine residues are displayed in ball and stick format and are colored red. (DOCX) [file pone.0229494.s004.docx]
