# Supplementary material for: Characterization and assembly of the Pseudomonas aeruginosa aspartate transcarbamoylase-pseudo dihydroorotase complex
Source: PLoS One. 2020 Mar 3;15(3):e0229494. doi: 10.1371/journal.pone.0229494 (PMC7053772; doi:10.1371/journal.pone.0229494)
Supplement: S5 Fig — Data for the carbamoyl phosphate, aspartate saturation curves and the inhibition by PALA. (DOCX) [file pone.0229494.s005.docx]

**S5 Fig. Effect of PALA the ATCase Activity of the ATCase-pDHO Complex**

Carbamoyl Phosphate (mM)

Specific Activity (μmol/min/mg)

0 nM PALA

1 nM PALA

25 nM PALA

500 nM PALA

100 nM PALA

PALA (nM) 0 1 25 100

V_max_ (μmol/min/mg) 108.8 ± 6.6 110.7 ± 5.4 78 ± 10.7 70.7 ± 4.3

K_m_ (S_0.5_) mM 1.27 ± 0.16 1.37 ± 0.12 1.74 ± 0.47 3.34 ± 0.24

n^H^ 1.46 ±0.20 1.65 ±0.20 1.4 ± 0.34 2.4 ± 0.24

χ^2^ 104.4 86.7 132.4 15.2

R 0.996 0.997 0.990 0.998
