# Supplementary material for: Characterization and assembly of the Pseudomonas aeruginosa aspartate transcarbamoylase-pseudo dihydroorotase complex
Source: PLoS One. 2020 Mar 3;15(3):e0229494. doi: 10.1371/journal.pone.0229494 (PMC7053772; doi:10.1371/journal.pone.0229494)
Supplement: S6 Fig — The inhibition of P. aeruginosa ATCase inhibition by PALA. Carbamoyl phosphate saturation curves of P. aeruginosa ATCase in the presence of 0 (●), 1 nM (○), 25 nM (■), 100 nM (□), 500 nM (X) of PALA was carried out as described in the text. The complex was formed by mixing stoichiometric concentrations of ATCase (6 μg) and pDHO (7.5 ug). The assay was conducted for 2 min at 37○ and 8 mM aspartate. The curves were fit using the program KaleidaGraph (Synergy Software) to the Hill equation: v = (S)nVmax/(Kmn+Sn) where n is the Hill coefficient. (DOCX) [file pone.0229494.s006.docx]

**S6 Table. Kinetic Data**

| **CP** |  | Activity |
| --- | --- | --- |
| (**mM)** |  | μmol/min/mg |
|  |  |  |
| 0.0 |  | 0.0 |
| 0.1 |  | 0.0 |
| 0.3 |  | 4.0 |
| 0.6 |  | 5.2 |
| 1.3 |  | 27.6 |
| 1.9 |  | 103.1 |
| 3.2 |  | 119.3 |
| 5.0 |  | 119.5 |
| 7.5 |  | 125.6 |

| **Asp** |  | **Actvity** |
| --- | --- | --- |
| mM |  | μmol/min/mg |
|  |  |  |
| 0.0 |  | 0.0 |
| 0.1 |  | 0.0 |
| 0.3 |  | 2.6 |
| 0.6 |  | 9.6 |
| 1.3 |  | 32.4 |
| 1.9 |  | 54.9 |
| 3.2 |  | 84.7 |
| 5.0 |  | 108.7 |
| 7.5 |  | 129.3 |
| 10.0 |  | 158.3 |
| 15.0 |  | 159.6 |

|  |  | **PALA (nM)** | |  |
| --- | --- | --- | --- | --- |
|  | **0.0** | **25.0** | **50.0** | **100** |
| **CP (mM)** |  |  |  |  |
| 0 | 0.0 | 0.0 | 0.0 | 0.0 |
| 0.06 | 0.0 | 0.0 | 0.0 | 0.0 |
| 0.32 | 10.8 | 4.0 | 0.0 | 0.0 |
| 0.60 | 32.5 | 21.4 | 4.5 | 1.6 |
| 1.25 | 49.7 | 26.3 | 8.8 | 8.9 |
| 1.90 | 66.9 | 37.1 | 14.6 | 12.1 |
| 3.15 | 91.9 | 60.0 | 27.6 | 33.9 |
| 5 | 97.2 | 65.2 | 35.4 | 51.1 |
| 7.5 | 98.0 | 66.2 | 61.1 | 61.6 |

Specific Activity (μmol/min/mg)
